# Embryo Culture Media Influence on Live Birth Rate and Birthweight after IVF/ICSI: A Systematic Review Comparing Vitrolife G5 Media to Other Common Culture Media

**DOI:** 10.5935/1518-0557.20200099

**Published:** 2021

**Authors:** Lena Bick, Anja Schulz Nielsen, Ulla Breth Knudsen

**Affiliations:** 1 Faculty of Health, Aarhus University, Nordre Ringgade 1, 8000 Aarhus, Denmark; 2 Department of Obstetrics & Gynecology, Regional Hospital Horsens, Sundvej 30, 8700 Horsens, Denmark

**Keywords:** Culture Media, Fertilization *in Vitro*, Reproductive Techniques, Assisted, Birth Weight, Pregnancy Rate, Live Birth

## Abstract

Previous studies have indicated that culture media vary in efficiency and outcomes, such as live birth rate, birthweight and embryo quality. Does Vitrolife G5 series culture media result in higher live birth rates and birthweight compared to other common culture media? This study is a systematic review based on the PRISMA criteria. Relevant search terms, mesh terms (PubMed and Cochrane) and Emtree terms (Embase) were identified. We searched the literature using PubMed, Embase and Cochrane, on November 10, 2019. The inclusion criteria involved published articles in English comparing Vitrolife G5 to other common culture media. We included randomized controlled trials (RCTs) and cohort studies. The quality of the studies was assessed using the Cochrane Risk of Bias tool 2.0 and the Newcastle-Ottawa Scale. Primary outcomes were live birth rate and birthweight. Secondary outcomes were fertilization rate, implantation rate, biochemical pregnancy rate, clinical pregnancy rate, miscarriage rate, multiple pregnancies and congenital malformations. Of 187 articles screened, 11 studies fulfilled the inclusion criteria: Five RCTs and six retrospective cohort studies. Only one study reported live birth rate, showing a non-significantly higher live birth rate for Vitrolife G5 media. Birthweight had equivocal results with three of six studies, showing significantly lower (2)/higher (1) birthweights, whereas the others were non-significant. Overall, there were no significant differences concerning secondary outcomes. The results are equivocal, and we need more studies to evaluate culture media and their effect on short- and long-term health.

## INTRODUCTION

In *in vitro* fertilization (IVF) and intracytoplasmic sperm injection (ICSI), the fertilized embryos are cultivated in culture media to choose the best embryo to transfer to the uterus either at cleavage stage or as a blastocyst. To make this possible, the oocytes and embryos are transferred to one or several culture media that support the early development of the embryos. These media have evolved from simple culture media based on blood serum to complex media containing a variety of different substances such as amino acids, human albumin, vitamins, antibiotics and growth factors (Chronopoulou & Harper, 2015).

While the culture media of the early years were homemade in fertilization clinics, fewer, but more specialized companies now commercially produce them. This has added economic interests, resulting in lack of transparency regarding media composition, but it has also led to increased quality and more quality control (Chronopoulou & Harper, 2015). Culture media can be divided into sequential media such as the G5 series (Vitrolife), where different culture media are used throughout the embryo development; or single media, such as GL BLAST sole medium (Ingamed), where only one single medium is used for the whole period, until the blastocyst stage.

Previous studies have indicated that different culture media vary in their efficiency and outcomes, such as live birth rate, birthweight and embryo quality (Youssef *et al*., 2015; Mantikou *et al*., 2013). Studies suggest that culture media influence gene expression and epigenetics in animals and humans, which might affect the long-term health of the children (Schwarzer *et al*., 2012; Kleijkers *et al*., 2015).

The number of infertile women submitted to IVF is increasing. Therefore, we undertook this study to compare the common culture media G5 series (Vitrolife, Sweden) to other common culture media, with the prime focus on live birth rates and birthweight.

## MATERIALS AND METHODS

We used the PRISMA criteria in this review. The study is registered in Prospero (CRD42020153820). Two of the review team members (L Bick and A S Nielsen) did data collection, data extraction and the assessment of the studies independently. Discussion or a third person (U B Knudsen) solved disagreements.

### Outcomes

The primary outcomes were live birth rate and birthweight. Live birth rate was defined as the proportion of women giving birth to at least one child born alive, independent of gestational age. Birthweight was defined as the mean birthweight of the babies measured in grams.

Secondary outcomes were fertilization rate, implantation rate, biochemical pregnancy rate, clinical pregnancy rate, miscarriage rate, multiple pregnancy rate and congenital malformations. Most definitions were based on Kleijkers *et al*. (2016), but may vary slightly among the different studies. The fertilization rate was defined as the percentage of fertilized oocytes (containing two pronuclei) among the number of mature oocytes (metaphase II) inseminated or injected. The implantation rate was defined as the number of gestational sacs identified by transvaginal ultrasound after six to eight weeks of gestation, divided by the number of embryos transferred. The biochemical pregnancy rate was defined as the percentage of women having at least one serum beta-hCG test of at least 50 UI/l two weeks after embryo transfer. The clinical pregnancy rate was defined as the percentage of women with a gestational sac and a fetal heartbeat, identified by transvaginal ultrasound examination at six to eight weeks of gestation. A miscarriage was determined as a biochemical pregnancy not resulting in a live birth. The multiple pregnancy rate was defined as the percentage of live births resulting in more than one child. Congenital malformations were divided into minor and major malformations. Major malformations were defined as malformations causing functional impairment or requiring surgical correction, and the remaining malformations were considered minor.

### Data Collection

We ran a systematic search on PubMed, Embase and the Cochrane Library on November 10, 2019.

The inclusion criteria were published articles in English on clinical trials containing well-defined data on at least one of the primary and/or secondary outcomes comparing Vitrolife G5 series culture media with other common culture media in humans. Both randomized controlled trials (RCTs) and cohort studies were included. Initially, there was no time limit on the search, but since Vitrolife G5 series was introduced in 2007, all articles from before 2007 were later excluded.

The research keywords was set up using the PICO model and divided into four search blocks. We used relevant search terms, mesh terms (PubMed and Cochrane) and Emtree terms (Embase). The four search blocks were used to run a combined search. The PICO table, search terms and examples of search queries can be found in the Supplements section of this review.

**Figure 1:**
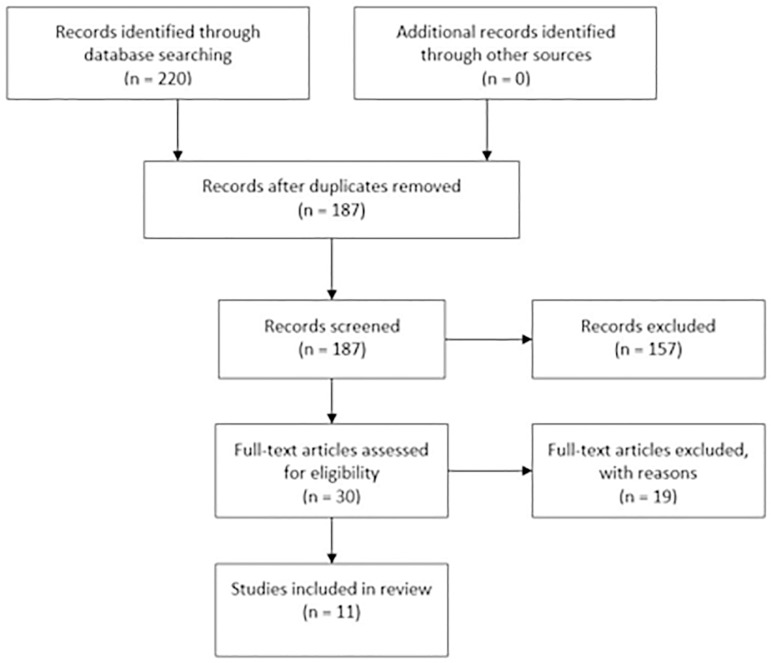
PRISMA flow chart.

The data collection is illustrated on the PRISMA flow diagram (Figure 1). The search in the three databases resulted in 44 results in PubMed, 63 results in Cochrane and 112 results in Embase. This yielded 219 results.

We removed the duplicates, resulting in 186 hits. We read the abstracts from the 186 results. When information was missing in the abstracts, for instance, whether the culture media was part of the Vitrolife G5 series, we searched for information in the full article. Of the 186 studies, 176 were excluded because either they did not contain any data comparing between Vitrolife G5 series and other culture media, they were animal studies, reviews, conference abstracts, or they were published before the G5 series was introduced in 2007. The participants in one study (Kleijkers *et al*., 2015) were enclosed in a larger study (Kleijkers *et al*., 2016), and therefore, only Kleijkers *et al*. (2016) was included. The reviews and their references were searched to find any data comparing G5 series media to other culture media.

We ran a Scopus citation search on the 10 remaining studies. The titles and abstracts of articles citing the 10 studies were searched to identify other relevant studies in which the systematic search might have been missing. One additional study was found, resulting in 11 studies to be included in this review.

### Data extraction and assessment of included studies

We read the included articles and extracted the data regarding primary and secondary outcomes. When available, we collected additional data, such as the type of G5 product, whether the study was an IVF/ICSI study, and whether the study used fresh or frozen embryos.

RCTs were assessed by Version 2 of the Cochrane risk-of-bias tool for randomized trials (RoB 2), shown in Table 1. The Newcastle-Ottawa Scale (NOS), shown in Table 3, assessed cohort studies. After the individual assessment was completed, a final assessment was found, and disagreements were solved by discussion or by a third person.

**Table 1:** Assessment of RCTs - Version 2 of the Cochrane risk-of-bias tool for randomized trials (RoB 2).

Reference	Domain 1	Domain 2	Domain 3	Domain 4	Domain 5	Overall Risk	Comments
Kleijkers *et al*. 2016							
Zhang *et al*. 2016							
Ceschin *et al*. 2016							
Hassani *et al*. 2013							
Hambiliki *et al*. 2011							No true randomization (alternate allocation)

Domain 1: Risk of bias arising from the randomization process

Domain 2: Risk of bias due to deviations from the intended interventions (effect of assignment to intervention)

Domain 3: Missing outcome data

Domain 4: Risk of bias in measurement of the outcome

Domain 5: Risk of bias in selection of the reported result

Risk of bias: green=low risk, yellow=some concerns, red=high risk

**Table 2:** RCTs - Table with additional information and comments. In the top the women were randomized, in the lower two studies the oocytes were randomized.

Reference	Country/year Multicenter/single-center	Randomization of women/oocytes	Number of participants	Comments
Kleijkers *et al*. 2016	The Netherlands, 2016 Multicenter	Women were randomized by a computer program	836 women, birthweight data from 360 children	Detailed description of methods used. Many participants. Blinding of couples, gynecologists, fertility doctors, outcome examiners. Intention to treat. Good description of handling dropouts. Power calculation included.
Ceschin *et al*. 2016	Brazil, 2016 Single-center	Women were randomly divided into two groups (not described in detail)	60 women, 311 mature oocytes for ICSI	Few participants. Acceptable description of methods, but short and not very detailed.
Hassani *et al*. 2013	Iran, 2013 Single-center?	Women were randomized before oocyte pick up according to a randomization list based on sequential numbers in sealed envelopes	538 women	Many participants. Good description of methods. Clear inclusion criteria of the women.
Zhang *et al*.,2016	China, 2016 Single-center	Oocytes were randomized according to a randomization table	37 women, 620 oocytes, 64 embryos transferred	Few participants. Good description of methods used. Focus is on early embryo cleavage kinetics.
Hambiliki *et al*.,2011	Sweden, 2011	Oocytes were divided to type of culture media via alternate allocation	110 women, 1206 oocytes, 108 embryo transfers	Many participants. Good descriptions of the methods with clear inclusion criteria and definitions of the outcomes.

**Table 3:** Assessment of retrospective cohort studies - Newcastle-Ottawa Scale (NOS)

Reference	Selection	Comparability	Exposure/Outcome	Total NOS-score	Comments
Lopéz-Pelayo *et al*., 2018	★★★★	-	★★★	7	
Gu *et al*. 2016	★★★★	★★	★	7	
De Vos *et al*., 2015	★★★★	★★	★★★	9	
Lin *et al*., 2015	★★★★	-	★★★	7	
Lin *et al*. 2013	★★★★	-	★★★	7	CPR only mentioned in laboratory protocol section
Eskild *et al*., 2013	★★★★	★★	★★★	9	

## RESULTS

The data collection is illustrated on the flow diagram (Figure 1). Of the 219 articles, only 11 studies qualified to be included in this review.

In Table 1, you find the RoB 2 assessment of the RCTs, and Table 2 shows additional information about the studies. Table 3 shows the NOS assessment of the retrospective cohort studies; and Table 4 shows additional information about the studies. Table 5 shows an overview of the 11 studies regarding the type of culture media, whether the study included IVF or ICSI or both, and which of the outcomes each study included. Table 6 shows the results of the primary outcomes, and Table 7 shows the results of the secondary outcomes.

**Table 4:** Retrospective cohort studies - Table with additional information and comments

Reference	Country/year Multicenter/single-center	Number of participants	Selection of participants and distribution of the culture media between the participants	Comments
Lopéz-Pelayo *et al*., 2018	Spain, 2018 Single-center	189 women	189 women undergoing infertility treatment at the center in 2016. The women were allocated to one of the culture media depending on the week of oocyte retrieval. The type of media was changed weekly.	A retrospective study with the fewest participants included in this review. Good description of the methods, inclusion criteria of the groups and definitions of outcomes.
Gu *et al*., 2016	China, 2016 Single-center	2370 singletons. 1755 cases from fresh embryo transfer and 615 from frozen embryo transfer.	Singletons born alive after 28 weeks of gestation who underwent IVF/ICSI cycles in the center between June 2009 and October 2012. Large proportion of embryos cultured in SAGE (SAGE 1336, Vitrolife 419). Gradual change from most embryos cultured in SAGE in 2009 and most embryos cultured in Vitrolife in 2012.	Large group of children. Good description of the methods. Good explanations about birthweight values, which are the focus of the study. Some of the values are adjusted for gestational age and gender. Pregnancies lost to follow up were excluded from data analysis. There is a risk that some of the babies are born to the same woman.
De Vos *et al*., 2015	Belgium, 2015 Single-center	2098 singleton live births resulting from only singleton pregnancies were included	Data was collected between April 2004 and December 2009. Medicult was used between April 2004 and April 2009. Vitrolife G3 was used from October 2004 and followed by G5 from September 2008 until December 2009.	The study has one table for birthweight showing the combined G3/G5 media compared to Medicult and a table comparing G3 to G5 media. Both are non-significant.
Lin *et al*., 2015	China, 2015 Single-center	8686 embryo cycles cultured in G5. 7706 embryo cycles cultured in G5 Plus. 7089 embryo cycles cultured in Global Medium.	Women who underwent IVF at the center between 2011 and 2013. One type of culture media was typically used for 3 days and then changed to another culture medium.	Large number of embryos. Clinical pregnancy rate is the only relevant outcome since focus is ectopic pregnancies in IVF-born children compared to spontaneous pregnancies. Not a good description of the distribution of the culture media, but it must be presumed that the embryos were cultured in only one of the three culture media, despite the change in media after three days. Data distinguishes between G5 and G5 Plus series.
Lin *et al*., 2013	China, 2013 Single-center	1201 singletons and 445 sets of twins	Women who underwent IVF at the center between 2008 and 2010. Singletons and twins born alive after 20 weeks of gestation. No information about the distribution and time of use of the different culture media at the center.	Large group of children. Good description of the methods. Multiple linear regression was performed to find confounding factors. No explanation about when the center used the different culture media.
Eskild *et al*., 2013	Norway, 2013 Single-center	2435 singletons	Singleton births from IVF/ICSI born after 22 weeks of gestation in the years 1999-2011. The culture media depends on the year: 1999-2007 Medicult Universal IVF Medium 2008-2009 Medicult Universal for fertilization and ISM1 for embryo culture 2009-2011 Vitrolife G-IVF Plus for fertilization and G-1 Plus for embryo culture.	Large group of children and data from many years. The focus is comparison between IVF children and spontaneous births. The comparison between the culture media is a comparison of different years where laboratory routines may differ. Linear regression was performed to find confounding factors. Adjustments were made for maternal age, number of previous deliveries and gestational age. There is a risk that some of the babies are born by the same woman.

**Table 5:** Culture media and outcomes overview of the different studies

Reference	G5 media, if specified	Other media, if specified	IVF/ICSI and fresh/frozen, if specified	LBR	BW	FR	IR	BPR	CPR	MR	MP	CA
Kleijkers *et al*., 2016	G-IVF Plus G-1 Plus G-2 Plus	HTF (Irvine Scientific)	IVF/ICSI Fresh/frozen	X	X	X	X	X	X	X	X	X
Zhang *et al*. 2016	G-IVF Plus G-1 Plus	Sequential media (Cook)	IVF Fresh			X	X		X			
Ceschin *et al*. 2016	G-1 Plus G-2 Plus	GV BLAST sole medium (Ingamed)	ICSI Fresh									
Hassani *et al*. 2013	G-1 and HAS EmbryoGlue	ISM1 (Medicult)	IVF/ICSI Fresh		X		X		X	X	X	
Hambiliki *et al*. 2011	G-IVF Plus G-1 Plus	Universal IVF medium/EmbryoAssist (Medicult)	IVF/ICSI Fresh?			X	X	X	X			
Lopéz- Pelayo *et al*. 2018	G-IVF G-1 plus G-2 plus	SAGE 1-STEP (Origio)	ICSI Fresh			X	X		X	X		
Gu *et al*., 2016	G5 HAS solution	Quinn’s advantage media (SAGE)	IVF/ICSI Fresh/frozen		X							
De Vos *et al*., 2015	G5	Universal IVF Medium, EmbryoAssist, and BlastAssist (Medicult)	IVF/ICSI Fresh		X							
Lin *et al*., 2015	G5 G5 Plus	Global (IVF Online)	IVF/ICSI Fresh						X			
Lin *et al*., 2013	G5 HSA solution	Quinn’s advantage media (SAGE) and Global culture medium (IVF online)	IVF Fresh		X				X			
Eskild *et al*., 2013	G-IVF Plus G-1 Plus	Universal IVF Medium and ISM1 (Medicult)	IVF/ICSI Fresh		X							

LBR: live birth rate, BW: birthweight, FR: fertilization rate, IR: implantation rate, BPR: biochemical pregnancy rate, CPR: clinical pregnancy rate, MR: miscarriage rate, MP: multiple pregnancies, CM: congenital malformations.

**Table 6:** Results, primary outcomes.

Live birth rate	G5 media	Result	Other media 1	Result	Other media 2	Result	*p*-value	S/NS
Kleijkers *et al*., 2016	G5	44.1	HTF	37.9			0.8	NS
**Birthweight**	**G5 media**	**Result**	**Other media 1**	**Result**	**Other media 2**	**Result**	***p*-value**	**S/NS**
Kleijkers *et al*., 2016	G5	Singletons+twins together Singletons:3299±46 Twins:2266±100 Fresh and frozen	HTF	158 lower in G5 Singletons:3480±44 Twins:2267±94 Fresh and frozen			0.008 0.005 0.99	S S NS
Hassani *et al*., 2013	G5	2660±80 fresh	ISM1	3030±70 fresh			0.001	S
Gu *et al*. 2016	G5	3196.0±468.9 Fresh 3300.6±441.3 Frozen	Quinn’s advantage medium	3168.4±462.0 Fresh 3256.0±466.7 frozen			0.29 0.27	NS NS
De Vos *et al*., 2015 *	G5/G3	3251±21 fresh	Universal IVF Medicult	3222±15 fresh			0.264	NS
Lin *et al*., 2013 ^†^	G5	3246.10±22.06 fresh 2500.63±30.74 fresh	Global	3293.88±26.26 fresh 2554.78±35.58 fresh	Quinn’s advantage medium	3291.24±43.45 fresh 2483.42±53.68fresh	0.327 0.397	NS NS
Eskild *et al*. 2013	G5	3441.4±637.2 fresh	Universal IVF Medium	3447.6±610.9 fresh	ISM1	3351.7±631.4 fresh	0.020	S

Live birth rate is measured in percent. Birthweight is measured in grams: mean ± SD. S/NS: significant/non-significant.

* Both G5 and G3 culture medium. 401 out of 710 are G5 culture medium.

† The first row shows results for singletons and the second row shows results for twins.

**Table 7:** Results, secondary outcomes.

Fertilization rate	G5 media	Result	Other media 1	Result	Other media 2	Result	*p*	S/NS
Kleijkers *et al*., 2016	G5	62.9	HTF	69.1			<0.001	S
Zhang *et al*., 2016	G5	71.3	Cook sequential medium	71.0			>0.05	NS
Ceschin *et al*., 2016	G5	67	GV BLAST sole	67			0.59	NS
Hambiliki *et al*., 2011	G5	73.5	Universal IVF Medium	67.2			0.030	S
Lopéz-Pelayo *et al*., 2018	G5	69.11	SAGE 1-STEP	70.07			0.736	NS
**Implantation rate**	**G5 media**	**Result**	**Other media 1**	**Result**	**Other media 2**	**Result**	***p***	**S/NS**
Kleijkers *et al*., 2016	G5	20.2 fresh	HTF	15.3 fresh			<0.001	S
Zhang *et al*., 2016	G5	29.0	Cook sequential medium	30.3			>0.05	NS
Hassani *et al*., 2013	G5	12	ISM1	15			0.16	NS
Hambiliki *et al*., 2011	G5	40.9	Universal IVF Medium	37.5			0.818	NS
Lopéz-Pelayo *et al*., 2018	G5	25.57	SAGE 1-STEP	30.16			0.520	NS
**Biochemical pregnancy rate**	**G5 media**	**Result**	**Other media 1**	**Result**	**Other media 2**	**Result**	***p***	**S/NS**
Kleijkers *et al*., 2016	G5	56.6	HTF	50.1			0.06	NS
Ceschin *et al*., 2016	G5	41.17	GV BLAST sole	38.46			0.83	NS
Hambiliki *et al*., 2011	G5	49.3	Universal IVF medium/EmbryoAssist	50.0			1.00	NS
**Clinical pregnancy rate**	**G5 media**	**Result**	**Other media 1**	**Result**	**Other media 2**	**Result**	***p***	**S/NS**
Kleijkers *et al*., 2016	G5	47.7	HTF	40.1			0.03	S
Zhang *et al*., 2016	G5	50.0	Cook sequential media	46.7			>0.05	NS
Hassani *et al*., 2013	G5	27.6	ISM1	32.1			0.23	NS
Hambiliki *et al*., 2011	G5	46.4	Universal IVF Medium	36.4			0.467	NS
Lopéz-Pelayo *et al*., 2018 *	G5	41.05 (37.7)	SAGE 1-STEP	55.88 (49.60)			0.213 (0.357)	NS
Lin *et al*., 2015 ^†^	G5	44.43	G5 Plus	43.34	Global	41.25		S
Lin *et al*., 2013	G5	42.9	Global	40.8	Quinn’s advantage medium	39.3		NS
**Miscarriage rate**	**G5 media**	**Result**	**Other media 1**	**Result**	**Other media 2**	**Result**	***p***	**S/NS**
Kleijkers *et al*., 2016	G5	15.8	HTF	13.4			0.33	NS
Hassani *et al*., 2013	G5	21.1	ISM1	20.5			0.9	NS
Lopéz-Pelayo *et al*., 2018 *	G5	9.52 (9.61)	SAGE 1-STEP	14.29 (16.90)			0.472 (0.266)	NS
**Multiple Pregnancy rate**	**G5 media**	**Result**	**Other media 1**	**Result**	**Other media 2**	**Result**	***p***	**S/NS**
Kleijkers *et al*., 2016	G5	10.3	HTF	13.2			0.40	NS
Hassani *et al*., 2013	G5	3.8	ISM1	8.5			0.19	NS
**Congenital malformations**	**G5 media**	**Result**	**Other media 1**	**Result**	**Other media 2**	**Result**	***p***	**S/NS**
Kleijkers *et al*., 2016	G5	Singletons: 2.5 Major 3.7 Minor Twins: 2.6 Major 2.6 Minor	HTF	Singletons: Major 4.4 Minor 4.4 Twins: 4.8 Major 0.0 Minor			0.52 0.78 1.00 0.48	Overall NS

All results are measured in percentages.

* Without brackets: Fresh. In brackets: Numbers for cumulative fresh and frozen ICSI.

† G5 and G5 Plus compared to Global. Significantly higher clinical pregnancy rates in the G5 and G5 Plus group compared to the Global group.

### Primary outcomes

#### Live birth rate

Kleijkers *et al*. (2016) found in an RCT that G5 culture media tended to have a slightly higher live birth rate than the HTF culture media, but the difference was non-significant (Table 6). None of the other studies report on live birth rate.

#### Birthweight

Two studies out of six found a significantly lower birthweight for G5 media, whereas one study found a significant higher birthweight for G5 (Table 6). Kleijkers *et al*. (2016) found in an RCT that G5 had a 158g lower birthweight compared to the HTF culture media; and Hassani *et al*. (2013) found in an RCT a 370g lower birthweight comparing G5 to ISM1. Eskild *et al*. (2013) found in a retrospective study a significant higher birthweight comparing G5 to Universal IVF medium and ISM1, where G5 was found to have a 92.4 g higher birthweight compared to ISM1.

Three of the retrospective cohort studies did not find any differences (Gu *et al*., 2016 - Quinn’s media, De Vos *et al*., 2015 - Medicult and Lin *et al*., 2015 - Global culture media), even though all three studies included more than one thousand embryos.

Kleijkers *et al*. (2016) included both fresh and frozen embryos in their analysis with total numbers only. De Vos *et al*., 2015 included both fresh and frozen embryos, and had separate results. The other studies included fresh embryos only.

### Secondary outcomes

#### Fertilization rate

One RCT study reported that G5 had a significantly lower fertilization rate compared to the HTF culture media (Kleijkers *et al*., 2016), and one RCT study reported that G5 had a significantly higher fertilization rate compared to Universal IVF Medium (Hambiliki *et al*., 2011) (Table 7). Two RCTs and a retrospective cohort study reported no differences comparing G5 to Cook Sequential Medium, GV Blast Sole and SAGE 1-STEP (Zhang *et al*., Ceschin *et al*., 2016; Lopez-Pelayo *et al*., 2018).

#### Implantation rate

One RCT found a significantly higher implantation rate for G5 compared to the HTF culture media (Kleijkers *et al*., 2016) (Table 7).

Three RCTs and a retrospective cohort study found no difference comparing G5 to Cook Sequential Medium, ISM1, Universal IVF Medium and SAGE 1-STEP (Zhang *et al*., 2016 Hassani *et al*., 2013; Hambiliki *et al*., 2011 Lopez-Pelayo *et al*., 2018).

#### Biochemical pregnancy rate

In three RCTs, no difference in biochemical pregnancy rate was found comparing G5 to HTF, GV Blast Sole and Universal IVF Medium (Kleijkers *et al*., 2016; Ceschin *et al*., 2016; Hambiliki *et al*., 2011) (Table 7).

#### Clinical pregnancy rate

In an RCT and in a retrospective cohort study, a significantly higher clinical pregnancy rate was found comparing G5 to HTF and Global (Kleijkers *et al*., 2016; Lin *et al*., 2015) (Table 7). Five studies including three RCTs and two retrospective cohort studies found no difference comparing G5 to Cook Sequential Media, ISM1, Universal IVF Medium, SAGE 1-STEP medium, Global, and Quinn’s advantage medium (Zhang *et al*., 2016; Hassani *et al*., 2013; Hambiliki *et al*., 2011; Lopez-Pelayo *et al*., 2018; Lin *et al*., 2013).

#### Miscarriage rate

In two RCTs and in a retrospective cohort study, no difference was found in miscarriage rate comparing G5 to HTF, ISM1 and SAGE 1-STEP media (Kleijkers *et al*., 2016; Hassani *et al*., 2013; Lopez-Pelayo *et al*., 2018) (Table 7).

#### Multiple pregnancy rate

Two RCTs found no difference in multiple pregnancy rates comparing G5 to HTF and ISM1 (Kleijkers *et al*., 2016, Hassani *et al*., 2013) (Table 7). The calculation of the percentages for Kleijkers *et al*. (2016) can be found in the Supplements section of this review.

#### Congenital malformations

Only one study reported on congenital malformations. In an RCT, no difference in numbers of congenital malformations was found comparing G5 and HTF media (Kleijkers *et al*., 2016) (Table 7).

## DISCUSSION

Defining the best embryo culture media can be a challenge as there are many different outcomes to assess the quality of the culture media. However, it is commonly accepted that live birth rate is the preferable outcome to assess IVF/ICSI success rates (Mantikou *et al*., 2013). Kleijkers *et al.* (2016) is the only study that evaluated live birth rate comparing G5 to another media, and they found a slightly higher live birth rate for G5 compared to HTF media, however not significant. The study was designed to detect a difference of 10%, but even a smaller difference may be of interest if this can be confirmed in more RCTs. The fact that only one of the studies included live birth rates (Kleijkers *et al*., 2016), which is considered the golden standard, clearly emphasizes the lack of RCTs reporting on live birth rate.

Some of the other studies had outcomes that approached live birth rates. Hambiliki *et al*. (2011) assessed delivery rate defined as the ratio between deliveries and embryos transferred. However, there are different guidelines for the numbers of embryos transferred per cycle. This makes comparison among centers difficult. Hassani *et al*. (2013) compared “baby take home rates” but gave no clear definition of the term. Future studies should adhere to the same definitions, and use live birth rate as the main outcome, so studies can be compared.

In this review, six of the eleven studies assessed birthweight with varying results. This is in line with previous studies, where some have shown that the type of culture media could influence birthweight (Dumoulin *et al*., 2010; Nelissen *et al*., 2012), other studies found no differences (Eaton *et al*., 2012; Vergouw *et al*., 2012). Birthweight is a popular outcome, but it is associated with several potentially confounding factors and it is complicated to interpret regarding the health of the child. On the contrary, larger birthweight might result in a higher risk of caesarian section, fetal hypoxia and stillbirth (Berntsen & Pinborg, 2018) and there may be later health risks for the child (Pinborg, 2019).

As mentioned, altering epigenetics is believed to be a mechanism that may be influenced by different culture media, and therefore might influence birthweight and future health of the child (Kleijkers *et al.*, 2015).

Some of the included studies assessed fresh embryo transfers only, while other studies assessed both fresh and frozen embryo transfers. Previous studies suggest the use of either fresh or frozen embryos could influence perinatal outcomes, and frozen embryo transfers might result in a higher birthweight than fresh embryo transfers (Wong *et al*., 2017; Berntsen & Pinborg, 2018). This is supported by the results on birthweight from Gu *et al*. (2016); and therefore, birthweight should be related to whether the child was the result of fresh or frozen embryo transfer.

The comparison between G5 series and other culture media is complicated by the fact that the Vitrolife G5 series consists of more than ten products according to their brochure (A link to the list of Vitrolife G5 products can be found in the references). Even inside the G5 series, there are different options for embryo culture media: G-1 Plus and G-2 Plus are ready for use, while addition of human serum albumin is needed in the equivalent G-1 and G-2. Previous studies suggest that these two options of protein sources inside the G5 series might result in a difference in birthweight (Zhu *et al*., 2014).

There were no significant findings in fertilization rate, biochemical pregnancy rates, miscarriage rates, multiple pregnancy rates and congenital malformations. The secondary outcomes must be interpreted with care regarding the quality of embryo culture media. Like birthweight, they become relevant if there is a clear correlation to IVF success rates, such as measured in live birth rates or child’s health.

While some of the media are sequential (G5 (Vitrolife), Sequential media (Cook), ISM1 (Medicult) and Quinn’s advantage media (SAGE), others are continuous/single media (HTF (Irvine Scientifics), GL BLAST sole medium, Universal IVF Medium (Medicult), SAGE 1-step (Origio) and Global (IVF online). No difference was found between single versus sequential media, which is in line with results from systematic reviews on this aspect (Sfontouris *et al*., 2016; Dieamant *et al*., 2017).

In general, the comparison of the studies is difficult since there are varying definitions of inclusion criteria for women, varying definitions of outcomes and varying laboratory routines. For instance, different guidelines for transferring one or more embryos at a time could influence some of the outcomes and may increase live birth rates. Most of the studies did not report on dropouts. It is unclear whether there were no dropouts or if they did not include them in their analyses and this might cause bias. In one retrospective cohort study (Lin *et al*., 2013), there were no clear descriptions of when they used one culture media or the other. If the distribution of the culture media is not random, this might cause selection biases. Only one study (Kleijkers *et al*., 2016) described a proper blinding in their methods. The lack of good description of the randomization between the culture media might be a problem. While some of the outcomes such as the biochemical pregnancy rates are measurable facts, there is a considerable subjectivity in the assessment of the best embryo for transfer.

There are some limitations to this review. There were five RCTs and six retrospective cohort studies. Only one RCT had a description of a good design including blinding of both patients and doctors, proper description and handling of dropouts and a power calculation. The other studies had varying data quality due to the description of the population, the randomization, handling of dropouts etc. Most studies randomized women, whereas in two studies the oocytes were randomized. As long as the randomization is done properly (and blinded), and the study has a reasonable size, this will most likely not influence the results. G5 media is compared to different culture media, which means that there are only few results examining some of the same outcomes (Table 6 and 7). The results of this review are based on comparing the results of the individual studies. Since the culture media, the inclusion criteria for the women and the definitions of the outcome vary in the studies; it was not possible to do a metanalysis on the topic.

As mentioned, there are many culture media available and many different outcomes, and so far there is very limited good evidence when comparing different culture media. This review indicates that no culture media is clearly superior or inferior to others, which allows the embryologist to take other factors such as affordability, availability, workload in the laboratory and experience/preference into account when choosing a media. Furthermore, the different outcomes highlight the importance of further research into media effects, both on success rates and on the long-term health issues, where evidence hopefully becomes available during the next years.

## CONCLUSION

In conclusion, Vitrolife G5 series culture media was found to have a trend towards higher live birth rates, but not significant compared to other common culture media. This result is comprised of only one trial (RCT).

Birthweight had equivocal results with three out of six studies showing significantly lower (2)/higher (1) birthweights, whereas the others were non-significant. Likewise, overall no significant differences were found concerning the secondary outcomes.

More RCTs are needed, with uniform definitions of outcomes. There is a lack of studies reporting on live birth rate. Most importantly, an effort should be made to assess culture media regarding the effect on short-term and long-term health of the IVF children.

## Supplements

Websites:

Link to the list of Vitrolife G5 products (last assessed April 15, 2020): http://www.evolutionvision.co.in/downloads/g5.pdf

Pico model

**Table:**

	Description	Search terms	PubMed and Cochrane mesh terms	Embase Emtree terms
Population	Infertile women attending IVF	IVF, in vitro fertilization, infertility, ART, assisted reproductive technology	“Fertilization in vitro”, “infertility”, “Reproductive Techniques, Assisted”	“In vitro fertilization”, “infertility”, “infertility therapy”
Indicator	Influence of culture media	Culture media, culture medium, culture system, embryo culture	“Culture media”	“Culture medium”
Comparison	Vitrolife G5 medium compared to other media	Vitrolife, G5, v5, G-1, G-2, G1, G2		
Outcome	Primary: live birth rate (LBR), birth weight (BW) Secondary: fertilization rate, implantation rate, biochemical pregnancy rate, clinical pregnancy rate, miscarriage rate, multiple pregnancy rate, congenital malformations	Live birth rate, birth rate, birth weight, birthweight Fertilization rate, implantation rate, biochemical pregnancy rate, clinical pregnancy rate, pregnancy rate, miscarriages, abortions, multiple pregnancies, congenital malformations, congenital abnormalities	“Birth rate”, “birth weight” “Pregnancy outcome”, “pregnancy, multiple, “congenital abnormalities”	Birth rate, birth weight Multiple pregnancy, pregnancy outcome,

### Searches

Pubmed search

First, the search terms were divided into PICO search blocks:

**P**: IVF OR in vitro fertilization OR infertility OR ART OR assisted reproductive technology OR "Fertilization in Vitro"[Mesh]) OR "Infertility"[Mesh] OR "Reproductive Techniques, Assisted"[Mesh]

**I**: Culture media OR culture medium OR culture system OR embryo culture OR "Culture Media"[Mesh]

**C**: Vitrolife OR G5 OR v5 OR G-1 OR G-2

**O**: Live birth rate OR birth rate OR birth weight OR birthweight OR Fertilization rate OR implantation rate OR biochemical pregnancy rate OR clinical pregnancy rate OR pregnancy rate OR miscarriages OR abortions OR multiple pregnancies OR congenital malformations OR congenital abnormalities OR "Birth Rate"[Mesh] OR "Birth Weight"[Mesh] OR "Pregnancy Outcome"[Mesh])OR "Pregnancy, Multiple"[Mesh] OR "Congenital Abnormalities"[Mesh]

The search blocks were then combined for the final search:

((((((((((((((IVF) OR in vitro fertilization) OR infertility) OR ART) OR assisted reproductive technology) OR "Fertilization in Vitro"[Mesh]) OR "Infertility"[Mesh]) OR "Reproductive Techniques, Assisted"[Mesh]))) AND (((((((Culture media) OR culture medium) OR culture system) OR embryo culture) OR "Culture Media"[Mesh])))) AND (((((((Vitrolife) OR G5) OR v5) OR G-1) OR G-2)))) AND (((((((((((((((((((((Live birth rate) OR birth rate) OR birth weight) OR birthweight) OR Fertilization rate) OR implantation rate) OR biochemical pregnancy rate) OR clinical pregnancy rate) OR pregnancy rate) OR miscarriages) OR abortions) OR multiple pregnancies) OR congenital malformations) OR congenital abnormalities) OR "Birth Rate"[Mesh]) OR "Birth Weight"[Mesh]) OR "Pregnancy Outcome"[Mesh]) OR "Pregnancy, Multiple"[Mesh]) OR "Congenital Abnormalities"[Mesh])))))

### Embase search

First, the search terms were divided into PICO search blocks:

**P**: IVF OR in vitro fertilization OR infertility OR ART OR assisted reproductive technology OR 'in vitro fertilization'/exp OR 'infertility'/exp OR 'infertility therapy'/exp

**I**: Culture media OR culture medium OR culture system OR embryo culture OR 'culture medium'/exp

**C**: Vitrolife OR G5 OR v5 OR G-1 OR G-2

**O**: Live birth rate OR birth rate OR birth weight OR birthweight OR Fertilization rate OR implantation rate OR biochemical pregnancy rate OR clinical pregnancy rate OR pregnancy rate OR miscarriages OR abortions OR multiple pregnancies OR congenital malformations OR congenital abnormalities OR 'birth rate'/exp OR 'birth weight'/exp OR 'pregnancy outcome'/exp OR 'multiple pregnancy'/exp OR 'congenital malformation'/exp

The search blocks were then combined for the final search:

(((ivf OR in) AND vitro AND fertilization OR infertility OR art OR assisted) AND reproductive AND technology OR 'in vitro fertilization'/exp OR 'infertility'/exp OR 'infertility therapy'/exp) AND ((((culture AND media OR culture) AND medium OR culture) AND system OR embryo) AND culture OR 'culture medium'/exp) AND (vitrolife OR g5 OR v5 OR 'g 1' OR 'g 2') AND (((((((((((live AND birth AND rate OR birth) AND rate OR birth) AND weight OR birthweight OR fertilization) AND rate OR implantation) AND rate OR biochemical) AND pregnancy AND rate OR clinical) AND pregnancy AND rate OR pregnancy) AND rate OR miscarriages OR abortions OR multiple) AND pregnancies OR congenital) AND malformations OR congenital) AND abnormalities OR 'birth rate'/exp OR 'birth weight'/exp OR 'pregnancy outcome'/exp OR 'multiple pregnancy'/exp OR 'congenital malformation'/exp)

### Cochrane

We ran the Cochrane search with the same search terms and mesh terms as the PubMed search.

### Calculation of multiple pregnancy rate

Calculation of multiple pregnancy rate for (Kleijkers *et al*., 2016):

A total of 383 live births were included: 165 singletons and 38 (17.8%) twins in the G5 group and 138 singletons and 42 (23.3%) twins in the HTF group. The multiple pregnancy rate was defined as the percentage of live births resulting in more than one child:

G5: 19/(19+165)=10.3%

HTF: 21/(21+138)=13.2%

The P-value was calculated to be 0.404 by using MEDCALC.

Link: https://www.medcalc.org/calc/comparison_of_proportions.php

### List of Vitrolife G5 products

List of G5 products according to a Vitrolife G5 series brochure. Name of the paper:

“The G5 Series™. Optimizing embryo development in a protective in vitro environment”

The brochure can be found on the following website: http://www.evolutionvision.co.in/downloads/g5.pdf

Products:

G-RINSE™

G-MOPS™ /G-MOPS™ PLUS

G-GAMETE™

G-IVF™ /G-IVF™ PLUS

G-1™ /G-1™ PLUS

G-2™ /G-2™ PLUS

EmbryoGlue^®^

G-PGD™

HSA-solution™

G-MM™

G-FreezeKit Blast™

G-ThawKit Blast™
